# Electrospun nanofibrous mats loaded with gemcitabine and cisplatin suppress bladder tumor growth by improving the tumor immune microenvironment

**DOI:** 10.1007/s10856-024-06786-y

**Published:** 2024-03-25

**Authors:** Jing Wang, Yisheng Yin, Xiang Ren, Shaogang Wang, Yunpeng Zhu

**Affiliations:** 1 Department and Institute of Urology, Tongji Hospital, Tongji Medical College, Huazhong University of Science and Technology, Wuhan, China; 2 Department of Thoracic Surgery, Tongji Hospital, Tongji Medical College, Huazhong University of Science and Technology, Wuhan, China

**Keywords:** Electrostatic spinning, Bladder cancer, Positive surgical margins, Topical chemotherapy, Tumor microenvironment

## Abstract

**Abstract:**

The perplexing issues related to positive surgical margins and the considerable negative consequences associated with systemic chemotherapy have posed ongoing challenges for clinicians, especially when it comes to addressing bladder cancer treatment. The current investigation describes the production of nanocomposites loaded with gemcitabine (GEM) and cisplatin (CDDP) through the utilization of electrospinning technology. In vitro and in vivo studies have provided evidence of the strong effectiveness in suppressing tumor advancement while simultaneously reducing the accumulation of chemotherapy drugs within liver and kidney tissues. Mechanically, the GEM and CDDP-loaded electrospun nanocomposites could effectively eliminate myeloid-derived suppressor cells (MDSCs) in tumor tissues, and recruit CD8^+^ T cells and NKp46^+^ NK cells to kill tumor cells, which can also effectively inhibit tumor microvascular formation. Our investigation into the impact of localized administration of chemotherapy through GEM and CDDP-loaded electrospun nanocomposites on the tumor microenvironment will offer novel insights for tackling tumors.

**Graphical abstract:**

**Figure:**
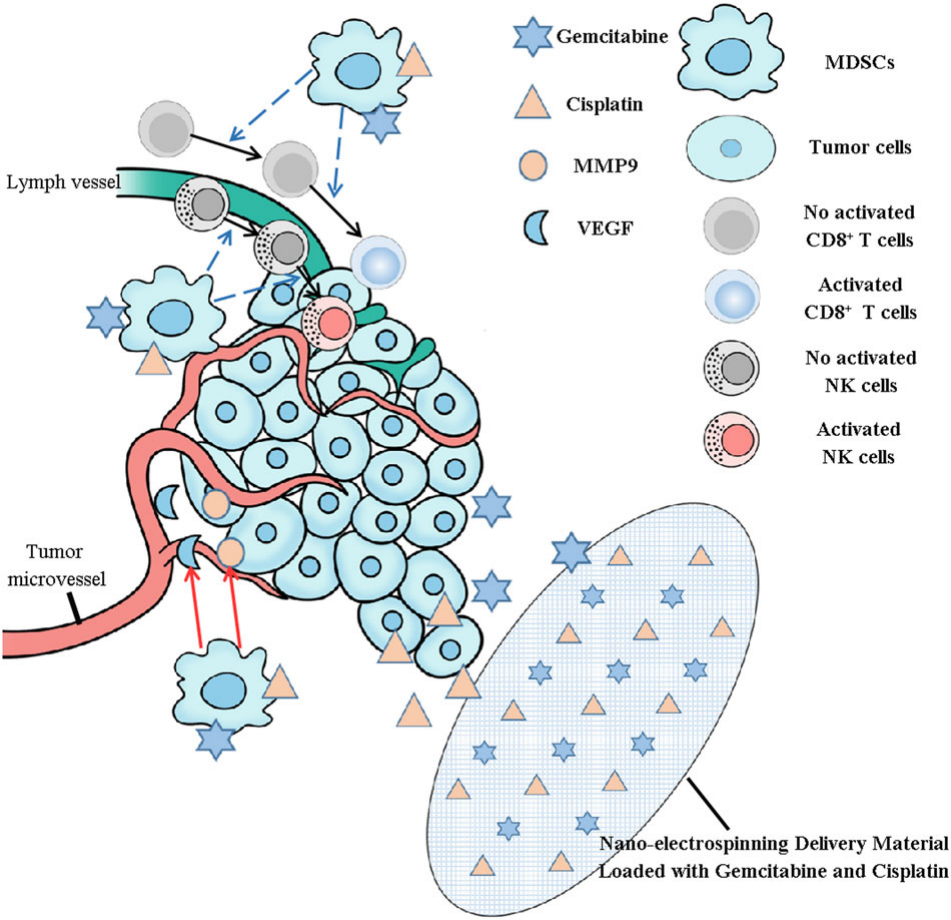
Drug-loaded electrospun nanofibrous mats suppress bladder tumor growth by improving the tumor immune microenvironment.

## Introduction

The incidence of tumors in various systems and organs has exhibited a substantial increase in recent years [1]. Recurrent growths in the form of distinct masses are commonly observed in solid tumors, and surgical intervention continues to be the foremost treatment approach employed in clinical settings [2–4]. Moreover, the challenges associated with positive surgical margins and postoperative tumor recurrence have long perplexed clinicians, particularly in the context of bladder cancer treatment [5, 6]. Bladder cancer is a prevalent malignancy in the genitourinary system, with non-muscle invasive bladder cancer accounting for 75–85% of all cases [7]. Transurethral resection of bladder tumor (TURBT) represents the cornerstone in the management of these patients. However, the recurrence rate after TURBT is significantly high, varying between 40 and 60% [8]. Hence, it is frequently necessary to administer regular intravesical chemotherapy in order to minimize the occurrence of tumor relapse following TURBT.

However, traditional intravesical chemotherapy is frequently administered with a short infusion time and low timeliness, which may predispose to urinary tract infection [9]. The development of a slow-release system for anti-tumor drugs introduces novel perspectives on postoperative chemotherapy, offering potential solutions to the challenges encountered in tumor chemotherapy after surgery [10, 11]. In the past decade, drug-controlled delivery systems have garnered significant attention due to their numerous advantages over conventional dosage forms, including enhanced drug delivery efficiency and reduced drug-related side effects [12–15]. Electrospun nano-sustained-release materials have been extensively investigated as a novel drug delivery platform, exhibiting immense potential in postoperative local chemotherapy within the realm of tumor growth inhibition and enhancement of the tumor microenvironment by enabling controlled release of various chemotherapeutic and immunotherapy agents [16–18].

The tumor microenvironment (TME) encompasses the extracellular matrix, immune cells, tumor microvessels, and diverse signaling molecules that surround tumor cells during tumorigenesis, growth, metastasis, and recurrence [19]. In the tumor microenvironment, myeloid-derived suppressor cells (MDSCs) were reported to promote tumor initiation, recurrence, and metastasis, making them one of the most crucial cell types involved in these processes [20, 21]. The localized delivery of drugs through electrospinning nano sustained-release materials can establish a continuous chemotherapy environment, thereby potentially inhibiting the growth of MDSCs and improving the local tumor microenvironment. This novel approach may offer a promising avenue for achieving long-term anti-tumor effects using nano-sustained-release materials, with no previous studies reported in this context.

In this study, we employed the classical electrospinning technique to fabricate a nanostructured sustained-release material using polylactic acid (PLA) as the raw material and incorporating the broad-spectrum anti-tumor drugs gemcitabine (GEM) and cisplatin (CDDP) for drug loading. The efficacy of this material was evaluated against bladder cancer as the target, focusing on its anti-tumor effect and impact on the tumor microenvironment.

## Materials and methods

### Fabrication of drug-loaded electrospun nanofibrous mats

The GEM/CDDP-loaded nanofibrous mats were fabricated via the electrospinning technique. Briefly, CDDP (0.1 mg) was dissolved in a dimethylformamide solution (1 ml), while GEM (0.2 mg) was dissolved in hexafluoroisopropanol (HFIP, 9 ml). These two solutions were mixed as the solvent, and polylactic acid (PLA, 1.7 g) was used as the solute. The solute was then thoroughly mixed with the solvent using a magnetic stirrer overnight to prepare the electrospun liquid. Subsequently, this electrospinning solution was loaded into an electrospinning machine (Ucalery, China), and the relevant parameters were set as follows: an electrostatic field voltage of 15 kV; an injection speed for the electrospinning liquid at 0.16 mm/min; and a distance between the syringe and receiving plate set at 18 cm. All experiments were conducted at room temperature (24 °C). Afterward, the resulting electrospun filament was placed into a low-temperature dryer and dried for 6 h. Finally, circular flakes with diameters of both 4 mm and 10 mm were obtained from this nano-sustained-release material using a hole puncher, followed by sterilization through low-temperature plasma sterilization.

The blank mats were prepared by dissolving 2.0 g of PLA in 10 ml of HFIP, following the same electrospinning parameters as mentioned above.

### Morphological analysis

The nanostructure of the sustained-release materials was observed using a scanning electron microscope (SEM). Prior to imaging, the samples were fixed onto metal stubs and coated with a thin layer of palladium–platinum–gold for 2 min to improve conductivity. The accelerating voltage of the SEM was set at 15 kV.

The ImageJ software (version 5.3, Bethesda, MD, USA) was utilized to determine the fiber diameters. The scale bar provided below each image served as a reference for calibrating the measurements of fiber diameter. Each individual fiber’s diameter was measured by drawing a line and recording it accordingly. A minimum of 50 measurements were taken in every micrograph, and the “average” function was used to calculate the mean value of fiber diameter.

### In vitro drug release study

Gemcitabine hydrochloride (National Institutes for the Food and Drug Control; China) standard solution with varying concentrations (0.25, 0.5, 1, 2, 4, 6, 8, 10, 20, 40, 60, and 80 μg/ml) was utilized. Fifteen circular mats with a diameter of 10 mm were randomly assigned into three groups and weighed before being immersed in PBS solution, normal saline solution, or triple-distilled water solution (50 ml each). The aforementioned standard curve was employed to determine the concentration of gemcitabine hydrochloride in different solvents after 1, 2, 3, 4, 5, 6, 7, 14, 21, 28, and 35 days of immersion. The high-performance liquid chromatography (HPLC) method was employed to determine the concentration of gemcitabine at different time intervals. Separation was accomplished using a C18 column measuring 4.6 cm in diameter and 150 mm in length, with a particle size of 5 μm. The mobile phase consisted of a mixture comprising ammonium acetate buffer (0.05 mol/l) adjusted to pH 5.7 using glacial acetic acid, along with methanol in a volume ratio of 90:10. Detection occurred at a wavelength of 269 nm.

### Anti-tumor effect of drug-loaded electrospun nanofibrous mats in vitro

The mouse bladder cancer cell line MB49 (National Collection of Authenticated Cell Cultures, Chinese Academy of Sciences) was utilized for both in vitro and in vivo studies. MB49 cells were cultured using RPMI-1640 medium (Gibco, USA) supplemented with 10% fetal bovine serum (Gibco). Well plates were used to seed the MB49 cells under optimal culture conditions, and they were divided into four groups for different treatments. The control group received no special treatment, while the PLA group served as the negative control and was treated with blank mats without drugs. The drug group acted as the positive control and received a combination of 0.8 mg GEM and 0.4 mg CDDP. The PLA-drug group was treated with nanofibrous mats loaded with GEM/CDDP (a 4 mm diameter electrospun material disc). To simulate drug metabolism in vivo, half of the medium was replaced every 24 h. After cultivation for 1, 2, or 3 days respectively, MB49 cells were collected for further analysis. Western blotting using an antibody against Caspase3 (Proteintech; China) was performed to assess tumor cell apoptosis. Flow cytometry utilizing the Annexin-FITC/PI Apoptosis Kit (Vazyme; China) was employed to determine the apoptosis rate.

### Anti-tumor effect of drug-loaded electrospun nanofibrous mats in vivo

**Fig. 1:**
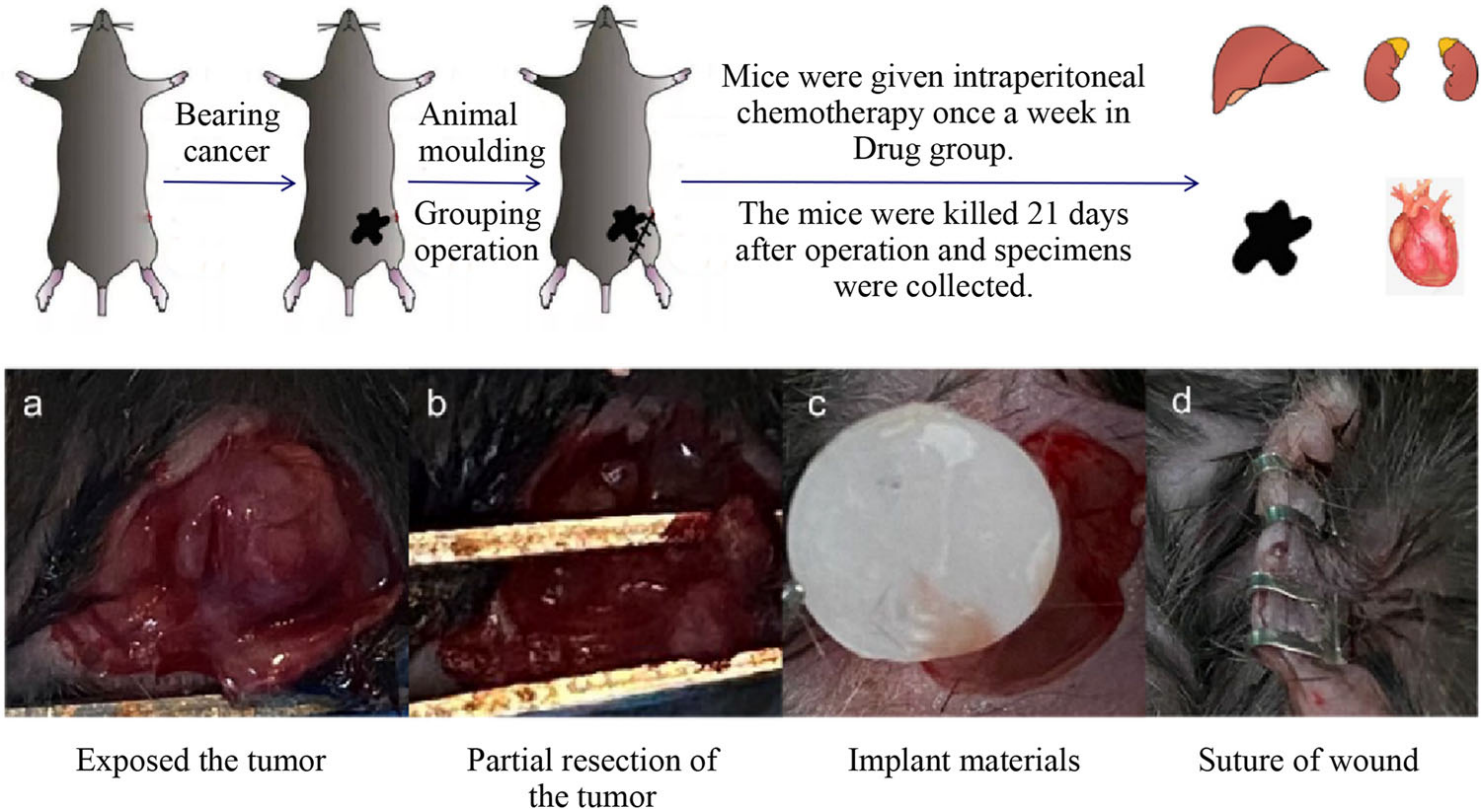
Design of the animal study. **a** Process of the animal study. **a**–**d** The procedure diagram

Thirty female C57BL/6 mice, aged 6 weeks, were procured from Hubei Bainte Biological Technology Co., LTD. MB49 cells in the logarithmic growth phase were harvested and prepared into a cell suspension with a concentration of 5 × 10^7^/ml. The C57BL/6 mice were anesthetized via inhalation, and a subcutaneous injection of 0.1 ml cell suspension was administered into the left groin region of each mouse. Approximately 1-week post-inoculation, visible nodules appeared in the subcutaneous tissue, and tumor diameter measurements were recorded daily thereafter. Mice with excessively large diameters (>13 mm), too-small diameters (<7 mm), or significant differences in body weight were excluded from further analysis. A skin incision measuring ~10 mm was made along the medial edge of the tumor to expose the subcutaneous tumor in suitable mice. Three-quarters of the tumor volume was excised to simulate positive surgical margins observed in human patients. Subsequently, these surgically treated mice were randomly divided into four groups: Control group—serving as a blank control group where wounds were directly sutured using wound clips without any specific treatment; PLA group—acting as a negative control group where two blank mats (4 mm diameter) devoid of drugs were placed at the incision site; Drug group—functioning as a positive control group where wounds were sutured using clips followed by intraperitoneal injections once per week with gemcitabine hydrochloride (0.04 mg/10 g) and cisplatin (0.02 mg/10 g); PLA-drug group—representing the experimental group wherein two sheets of electrospun nano sustained-release material (4 mm diameter) containing drugs were positioned at the incision site’s edge (Fig. 1). The body weight and tumor volume of mice were measured biweekly following the surgical procedure. Three weeks post-operation, liver, kidney, blood, and tumor tissue samples from the mice were collected. These tissue samples were subsequently stored in liquid nitrogen or formaldehyde fixative for preservation purposes. To investigate apoptosis in tumor tissues of each group, a TUNEL fluorescent apoptosis kit was employed.

### Biodistribution study

The content of GEM in mouse liver, kidney, blood, and tumor samples was determined using ultra-performance liquid chromatography-tandem mass spectrometry (UPLC-MS/MS) [22]. A methanol-water extract (volume ratio 3:1, 500 μl) was added to tissue samples (liver, kidney, tumor) obtained from mice in the Drug group and PLA-Drug group. The samples were then ground, vortexed, and subjected to ultra-high-speed centrifugation to obtain clear liquid. Methanol was used as the extraction solvent after vortexing, and the supernatant obtained after ultracentrifugation served as the sample. The Agilent 1290 Infinity II series (Agilent Technologies) ultra-performance liquid chromatography system with an Agilent ZORBAX Eclipse Plus C18 column (2.1 mm * 100 mm, 1.8 microns) was employed under the following conditions: aqueous phase consisting of 0.1% formic acid and organic phase composed of methanol solution. A standard curve based on GEM standards was established to calculate its concentration in different samples.

### Effect of drug-loaded electrospun nanofibrous mats on tumor microenvironment

The Gr-1 (antibody of Gr-1; BOSTER; China) and CD11b molecules (Antibody of CD11b; Abcam; America) were used. Double-label immunofluorescence staining was used to identify the distribution of MDSCs in tumor tissues. Immunohistochemical staining and fluorescence histochemistry were used to detect the expression of CD8 protein (Antibody of CD8; BOSTER; China), NKp46 protein (Antibody of NKp46; BOSTER; China), showing the distribution of CD8^+^ T cells and NKp46^+^ NK cells in tumor tissues. The expression levels of vascular growth-related proteins Flk-1, MMP9, and VEGF in tumor tissues were detected by Western Blot. CD34 was used as a marker (Antibody of CD34; Baiqiandu Biotechnology; China) to show the distribution of microvessels in tumor tissues by immunohistochemistry.

### Statistical analysis

The data were analyzed using SPSS software (Version 21.0; SPSS, USA). Descriptive statistics including mean ± standard deviation were used to analyze normally distributed data, while the median (Q1, Q3) was employed for non-normally distributed data. An Independent sample *t*-test was conducted to compare the differences between the two groups when the data followed a normal distribution. For non-normally distributed data, the Mann–Whitney *U* test was utilized to determine significant differences between groups. Statistical significance was defined as *p* < 0.05.

## Results

### Material characterization

**Fig. 2:**
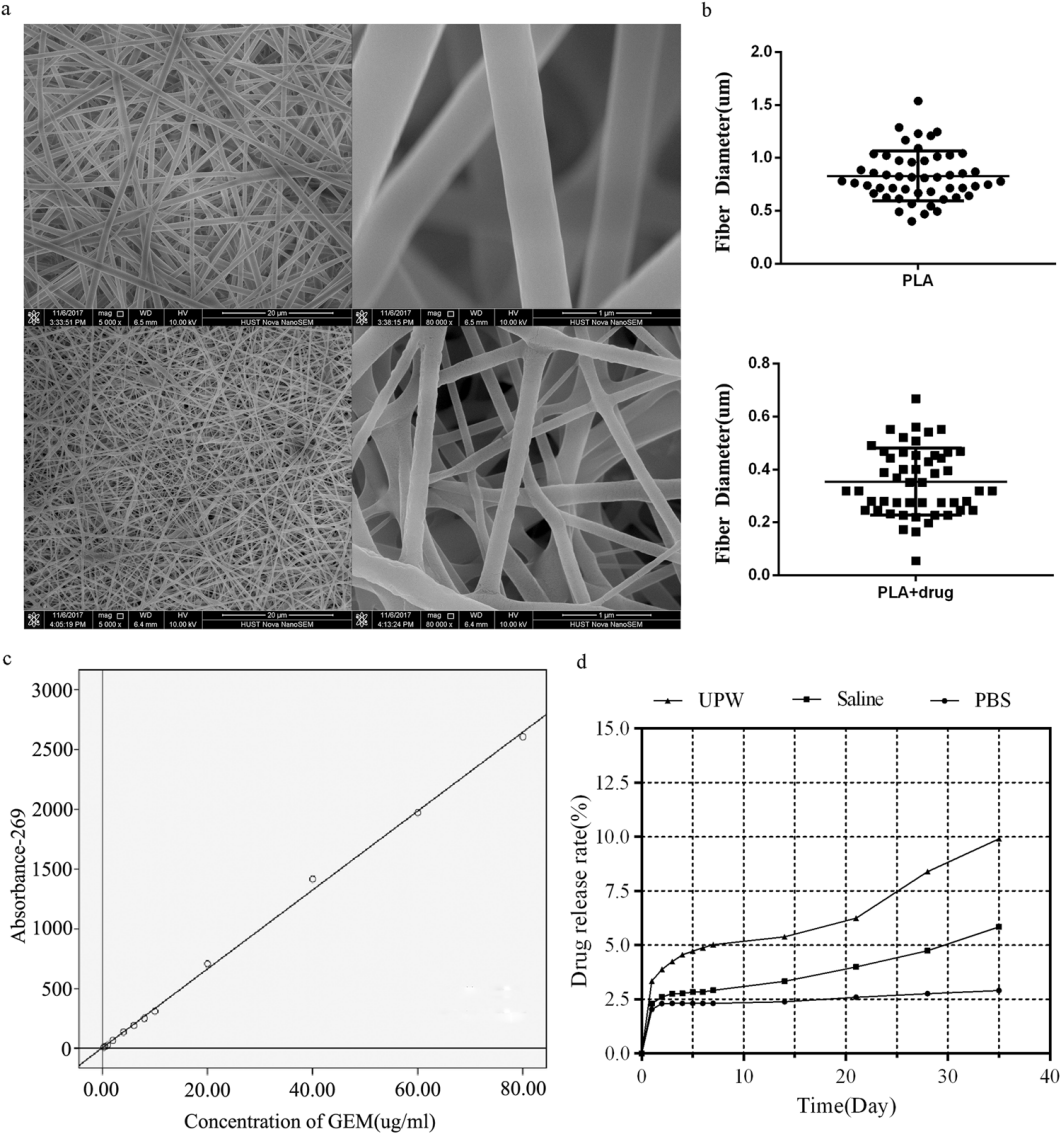
Properties of drug-loaded electrospun nanofibrous mats. SEM images of the surface of (**a**) PLA mat and PLA-drug mat and (**b**) quantitative analysis of the fiber diameter distribution. **c** The standard curve of GEM and absorbance. **d** The release profiles of the PLA and PLA-drug mats in various solutions

Under the scanning electron microscope, the crisscrossed nanofibers exhibited interconnectivity, forming a well-defined network structure. Each fiber displayed a sleek surface, consistent thickness, and absence of discernible defects (Fig. 2a). The quantitative analysis revealed that the average fiber diameter in the PLA mat and the PLA + drug mat was 364.25 ± 50.25 nm and 815.88 ± 83.56 nm, respectively (Fig. 2b).

The standard curve of GEM and absorbance was generated by measuring various concentrations of standard GEM: A (absorbance) = 0.0033*C (drug concentration) − 0.0286 (*R*^2^ = 0.998) (Fig. 2c). By analyzing the drug release profiles of the mats in various solutions, it is evident that a sustained and gradual drug release occurs over time. Notably, the drug-loaded nanofibers exhibit an initial burst release on day 1, with the most pronounced release observed in triple distilled water (Fig. 2d).

### Anti-tumor effect of drug-loaded electrospun nanofibrous mats in vitro

**Fig. 3:**
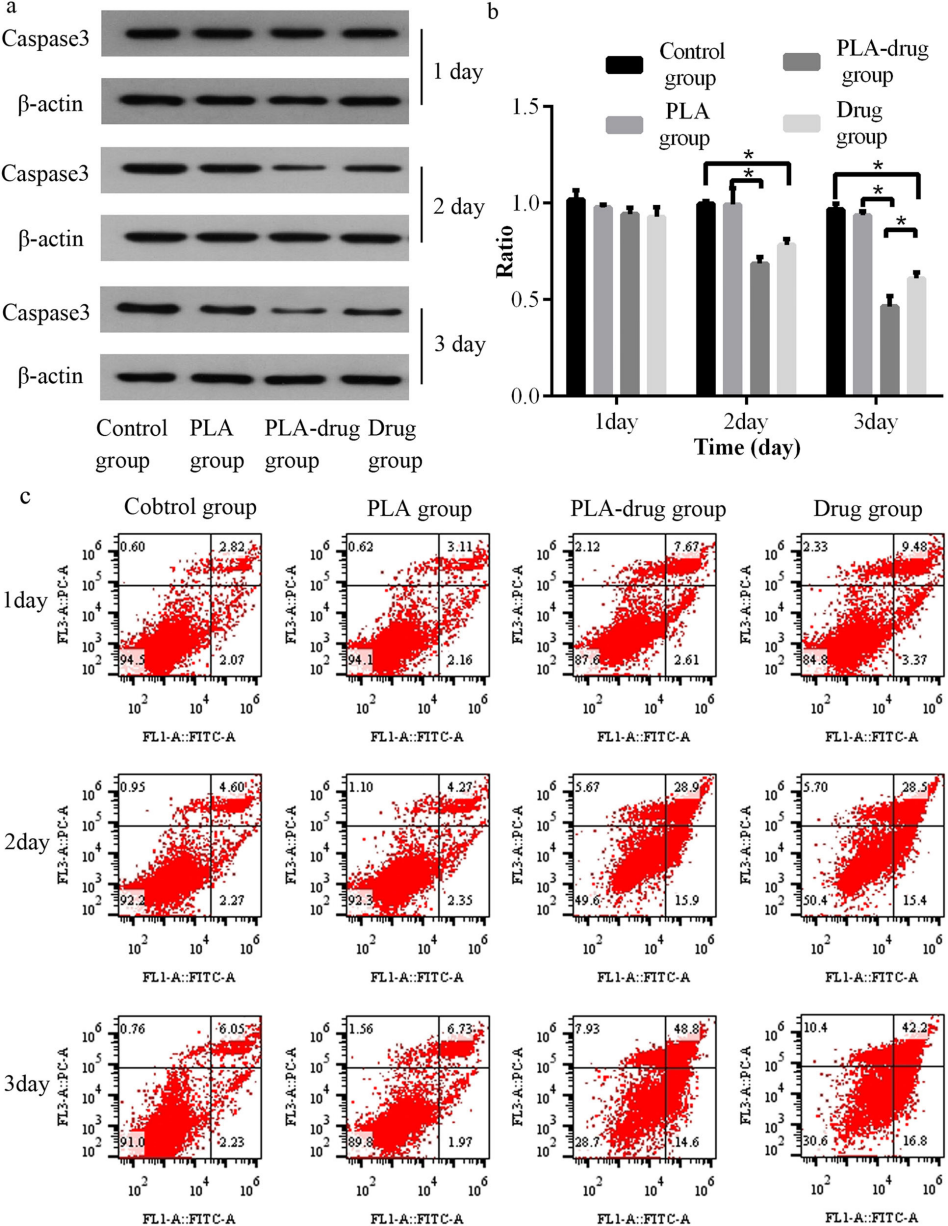
Study of the anti-tumor efficacy in vitro. **a** The western blotting presented the expression of Caspase3 of the MB49 cell and (**b**) quantitative analysis. **c** The apoptotic cell percentage was measured by flow cytometry. *p < 0.05

In the PLA-drug group, Caspase3 proteins in the tumor cells rapidly fell within 3 days. On the 2nd and 3rd day after treatment, the Caspase3 protein in the PLA-drug group was significantly lower than that in the PLA group (*p* < 0.005). On the 3rd day after treatment, the expression of Caspase3 protein in the tumor cells of the PLA-drug group was also significantly different from that of the Drug group (*p* = 0.046). As depicted in Fig. 3c, there was no significant alteration observed in the apoptosis rate within the Con group and PLA group throughout the observation period; however, a substantial increase in apoptotic cells was evident in both the drug-treated group and PLA-drug combination group. After the treatment with the drug-loaded mats for 3 days, the percentage of apoptotic cells in the PLA-drug group was higher.

### Anti-tumor effect of drug-loaded electrospun nanofibrous mats in vivo

**Fig. 4:**
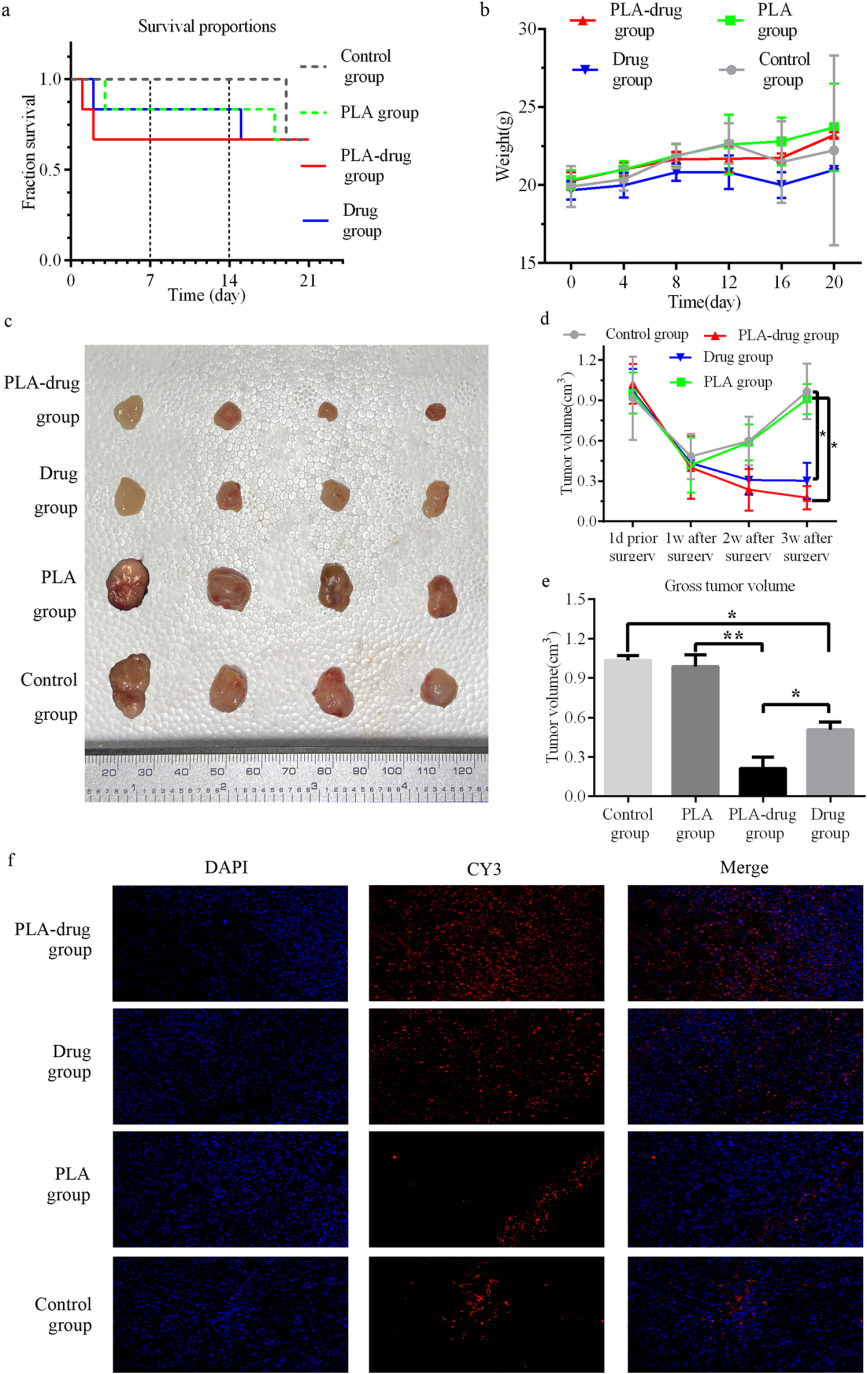
Study of the anti-tumor efficacy in vivo. The survival curve (**a**) and the (**b**) weight change curve. **c** Typical images of subcutaneous tumors. **d** The tumor volume change curve. **e** Quantitative analysis of the tumor volume. **f** Typical images of the TUNEL fluorescence staining of subcutaneous tumors. *p < 0.05, **p < 0.01

The survival curve and the weight change curve of the experimental animals were drawn (Fig. 4a, b). In the Control group, all mice succumbed on the 19th-day post-surgery due to extensive tumor growth and ascites formation, indicating tumor progression and metastasis as potential causes of mortality. In the PLA-drug group, two mice succumbed on the 1st and 2nd postoperative days. The mortality rate was predominantly observed within the initial 2 days after surgery in the PLA-drug group, potentially attributed to local reactions following slow-release material implantation. Weight measurements consistently demonstrated lower values in the Drug group mice compared to other groups, indicating gastrointestinal adverse effects of intravenous chemotherapy medication (accompanied by observable symptoms of diarrhea in the Drug group mice).

Tumor diameter was assessed every week following surgery, and tumor growth was determined by calculating the volume using the formula (volume = 0.52 * major diameter * minor diameter * minor diameter). At 3 weeks postoperatively, a reduction in tumor volume was observed in both the PLA-drug group and drug group of mice, while gradual increases in tumor volume were noted in the PLA group and Control group. The PLA-drug group exhibited a significantly reduced tumor volume compared to the PLA group in the third-week post-surgery (*p* = 0.011). The tumor volume of the Drug group mice was also significantly smaller than that of the Control group (*p* = 0.02) (Fig. 4d). At the third week after surgery, the tumor tissues were removed completely after the mice were euthanized. The volume of tumor tissue in each group was compared, and the results were similar to those described above in vitro (Fig. 4c). The tumor volume of the PLA-drug group was significantly smaller than that of the PLA group (*p* = 0.008). The tumor volume of the Drug group was significantly smaller than that of the Control group (*p* = 0.012) (Fig. 4e). These results indicated that both drug delivery and intraperitoneal administration could effectively inhibit the growth of bladder cancer tumors. Comparing the tumor volume of the Drug group and the PLA-drug group, we found that the tumor volume of the PLA-drug group was smaller (*p* = 0.041). This indicates that compared to the abdominal cavity, sustained sustained-release drug method may provide a better therapeutic effect.

Additionally, the apoptosis of tumor cells in the tumor tissues of each experimental group was visualized using TUNEL apoptosis fluorescence staining. A significantly higher percentage of tumor cell apoptosis areas, indicated by red fluorescence in the tumor sections, in both the PLA-drug and Drug groups compared to the Control and PLA groups. Furthermore, the area of red fluorescence was also found to be higher in the PLA-drug group than in the Drug group (Fig. 4f).

### Effects of drug-loaded electrospun nanofibrous mats on tumor microenvironment

**Fig. 5:**
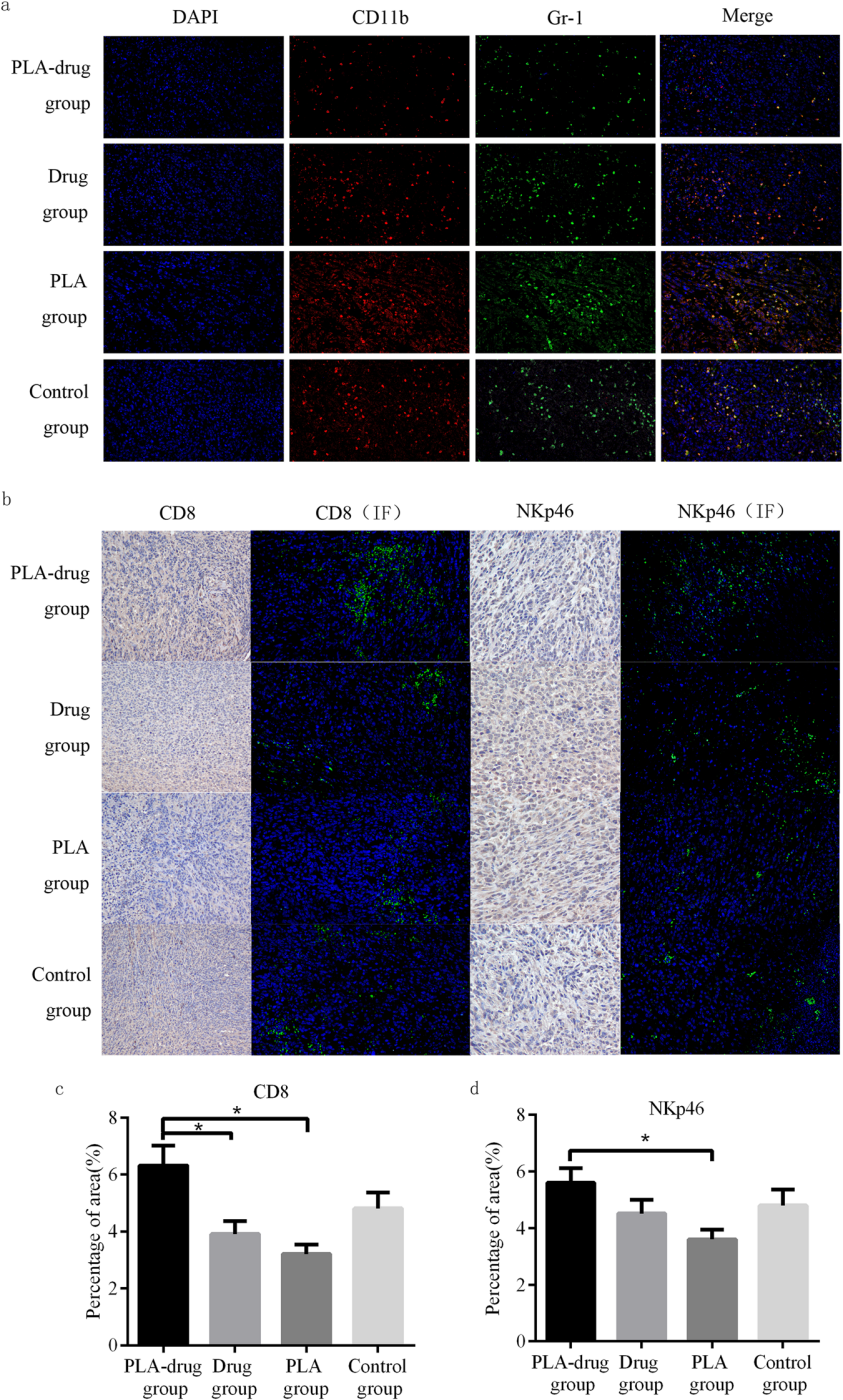
Study of effects on MDSCs. **a** Typical fluorescence images of the Gr-1 and CD11b. **b** Typical fluorescence images and immunohistochemistry images of the CD8 and NKp46 and quantitative analysis (**c**, **d**)

Gr-1 and CD11b double-labeled molecules were used to identify MDSCs and double-labeled cells in tissue sections were considered MDSCs. In the fluorescence pictures of the PLA-drug group, the number of MDSCs was significantly less than that of the other three groups, indicating that the slow-release drug administration can effectively reduce the number of MDSCs in tumor tissues (Fig. 5a).

We used CD8 protein to label CD8^+^ T cells and NKp46 protein to label NKp46^+^ NK cells. By immunohistochemistry, we found that CD8 protein (*p* = 0.008) and NKp46 protein (*p* = 0.026) in tumor tissues of the PLA-drug group were significantly higher than those of the PLA group. This indicated that the distribution of corresponding CD8^+^ T cells and NKp46^+^ NK cells in the tumor tissues of the PLA-drug group was higher than that of the PLA group (Fig. 5b).

**Fig. 6:**
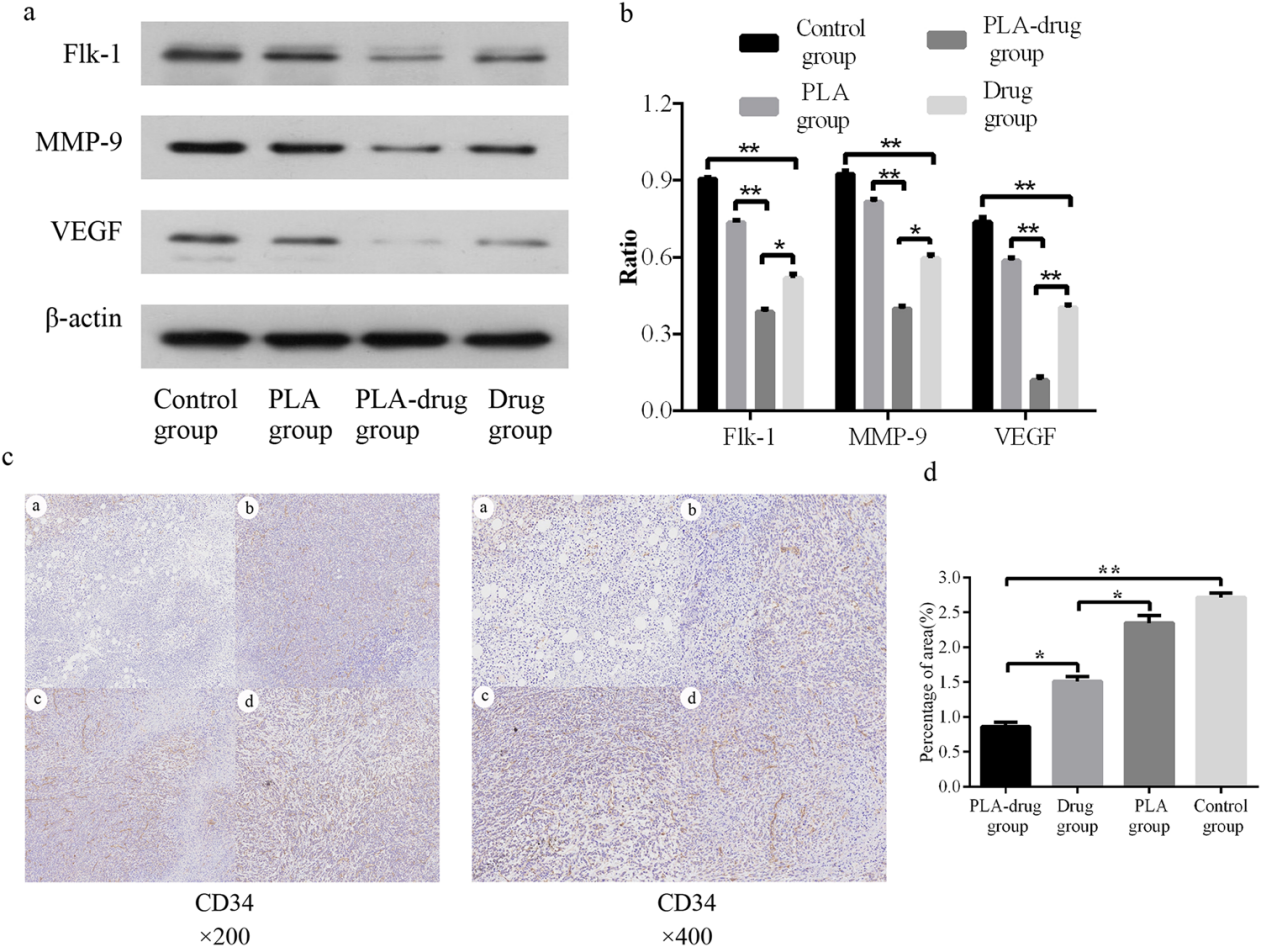
Study of effects on neovascularization. **a** Western blot showed the expression of Flk-1, MMP9, and VEGF, and (**b**) quantitative analysis. **c** Typical immunohistochemistry images of the CD34 and (**d**) quantitative analysis. *p < 0.05, **p < 0.01

The results of Western Blot showed that the expression levels of the neovascularization markers, Flk-1, MMP9, and VEGF, in the tumor tissues of the PLA-drug group were significantly lower than those of the other three groups (Fig. 6a, b). CD34 is a marker of vascular endothelial cells, which can show the distribution of blood vessels in tumor tissue. According to the results of CD34 immunohistochemical staining in tumor tissue, the PLA-drug group had a significant reduction in the expression of CD34 protein in tumor tissue compared with the other three groups (Fig. 6c, d). These results indicated that the number of microvessels in the tumor tissue of the PLA-drug group was significantly lower than that of the other three groups.

### Biodistribution study

**Fig. 7:**
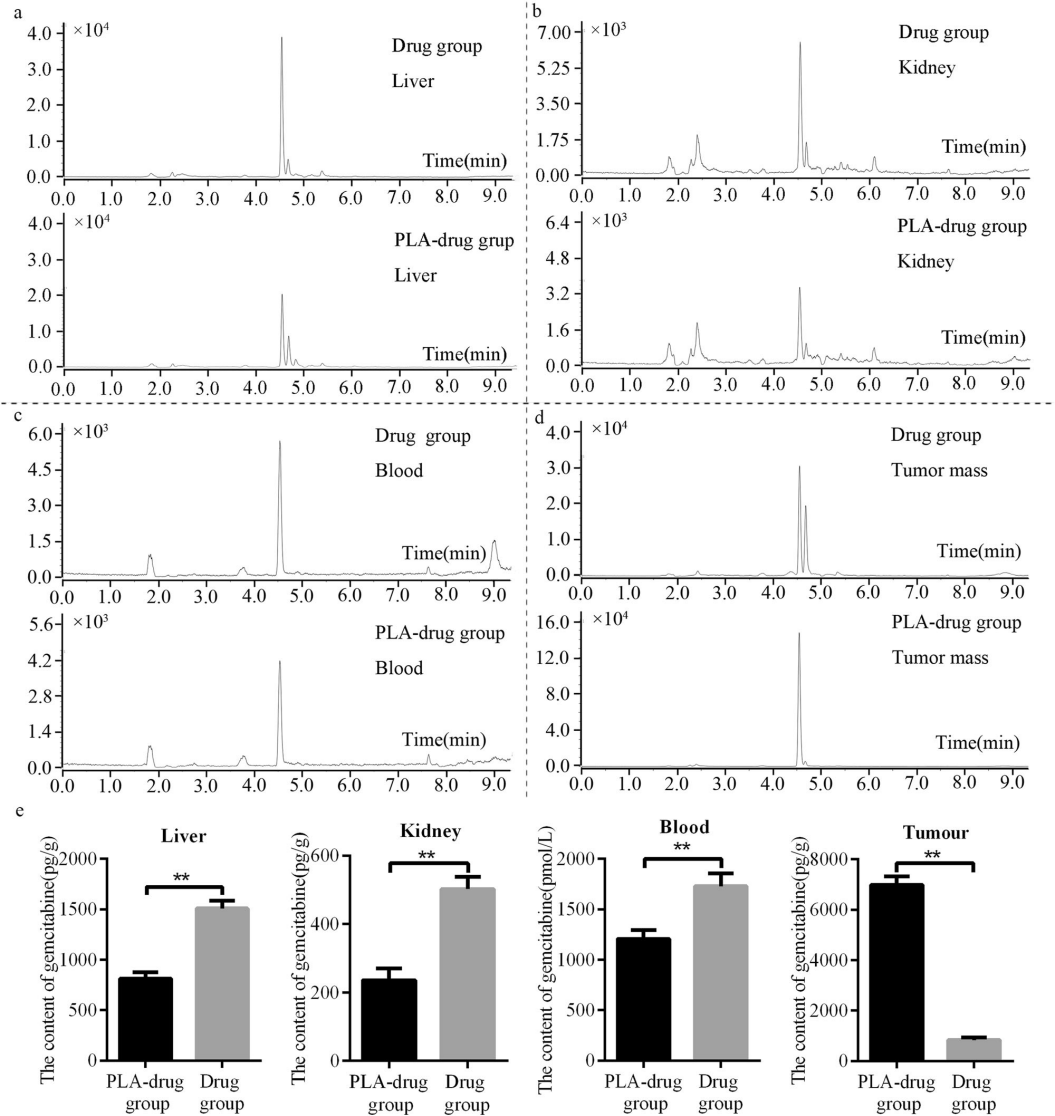
Biodistribution study. The accumulated amount of gemcitabine hydrochloride in (**a**) liver, (**b**) kidney, (**c**) blood, (**d**) tumor tissue, and (**e**) quantitative analysis. **p < 0.01

The accumulated amount of gemcitabine hydrochloride in the liver, kidney, blood, and tumor tissue of PLA-drug group and Drug group mice were measured by UPLC-MS/MS. We found that the content of gemcitabine hydrochloride in the liver samples of the Drug group was significantly higher than that of the PLA-drug group (1509.97 ± 176.42 vs. 813.48 ± 90.71 pg/g, *p* < 0.001) (Fig. 7a). The content of GEM in the Drug group was also significantly higher than that in the PLA-drug group (502.39 ± 55.41 vs. 235.52 ± 34.93 pg/g, *p* = 0.001) (Fig. 7b). This revealed that local delivery of sustained-release materials can effectively reduce the accumulation of chemotherapeutic drugs in the liver and kidney. The serum level of GEM in the Drug group was significantly higher than that in the PLA-drug group (1729.06 ± 126.33 vs. 1208.02 ± 88.73 pmol/l, *p* < 0.001) (Fig. 7c). In the tumor tissue, the content of GEM in the Drug group was significantly lower than that in the PLA-drug group (832.93 ± 79.14 vs. 6993.01 ± 337.08 pg/g, *p* < 0.001) (Fig. 7d). The concentration of GEM in the tumor tissue was only one-eighth in the Drug group compared to the PLA-drug group. This disparity likely accounts for the superior control of tumor volume observed in the PLA-drug group at 3 weeks post-surgery.

## Discussion

Tumor recurrence following surgical intervention and the significant adverse effects associated with systemic chemotherapy pose inevitable challenges in the management of solid tumors [23, 24]. The present study reported the fabrication of GEM and CDDP-loaded nanocomposites using electrospinning technology. In vitro and in vivo experiments have substantiated the potent efficacy in inhibiting tumor progression while concurrently mitigating chemotherapy drug accumulation within liver and kidney organs. Mechanically, the GEM and CDDP-loaded electrospun nanoparticles could effectively eliminate MDSCs in tumor tissues, and recruit CD8^+^ T cells and NKp46^+^ NK cells to kill tumor cells, which can also effectively inhibit tumor microvascular formation.

In the study in vitro, the apoptosis rate of tumor cells was significantly higher in the PLA-drug group compared to the PLA group, while it was similar to that in the drug group. Both slow-release material-mediated drug administration and direct drug administration effectively induce tumor cell death. This observation is further supported by the depletion of the Caspase3 protein. In the study in vivo, the tumor volume of the PLA-drug group and Drug group was significantly smaller than that of the control group. Moreover, the tumor volume of the PLA-drug group was significantly smaller than that of the Drug group. In the Tunel staining of tumor tissue, we also observed that the area of cell apoptosis in the PLA-drug group was significantly larger than that in the drug group. The results of the in vivo experiment also confirmed that the drug sustained-release nanomaterials had a better anti-tumor effect than intraperitoneal chemotherapy. Our findings align with previous research papers [25, 26], however, the majority of scholars substantiate the efficacy of novel anti-tumor materials through in vivo and in vitro experiments, while neglecting to delve into the underlying molecular mechanisms [27, 28].

Why does the drug sustained-release material have a more significant anti-tumor effect than intraperitoneal chemotherapy? On one hand, it is hypothesized that this phenomenon may be attributed to the distribution and metabolism of drugs [29]; on the other hand, it could potentially be ascribed to the substantial impact of slow-release materials on drug delivery within the tumor microenvironment [26]. Previous studies have demonstrated that local administration of sustained-release materials exhibits superior hepatoprotective, nephroprotective, and hematopoietic-preserving effects compared to intravenous administration [30, 31]. The local drug concentration within the tumor and the cumulative drug concentration in normal tissues (liver, kidney, and blood) were quantified in this study. The findings substantiate that localized administration of slow-release materials can significantly enhance drug delivery efficacy while mitigating chemotherapy drug accumulation in normal tissue. The findings of our study make a significant contribution to the existing body of research.

Through the immunofluorescence histochemistry, we observed that the distribution of MDSCs in the PLA-drug group was significantly reduced in the tumor tissues. At the same time, as the effector cells of MDSCs, CD8^+^ T cells and NKp46^+^ NK cells were significantly increased in the PLA-drug group. The utilization of T cells is rapidly gaining prominence as a promising therapeutic approach for the treatment of cancer and various other diseases. Alex et al. [32] proposed a materials-based strategy to enhance the activation and expansion of T cells, employing electrospinning techniques to fabricate a poly(ε-caprolactone) fiber mesh that serves as a platform for presenting activating ligands targeting CD3 and CD28 receptors, thereby promoting T cell activation and subsequent expansion. Our results suggested that MDSCs were effectively cleared from the tumor tissue due to the improvement of local chemotherapy delivery efficiency of the sustained-release materials, thereby restoring the normal recruitment and differentiation ability of CD8^+^ T cells and NKp46^+^ NK cells in the tumor tissue, enhancing the anti-tumor effect of the autoimmune system. Moreover, based on the study of angiogenesis, slow-release materials can also effectively inhibit tumor microvascular formation within the tissue, thereby further restricting tumor growth [33, 34].

Although our experiments have confirmed the chemotherapeutic effect of locally delivered nano-sustained-release materials and their impact on the immune microenvironment, there are still certain limitations in our study. Firstly, there is a lack of comprehensive research on the in vivo decomposition and release process of nano-sustained-release materials. Despite conducting relevant in vitro simulation experiments, they may not fully represent real-life scenarios. Secondly, due to the limited number of experimental animals and the relatively short duration of observation, it remains unclear whether nano-sustained-release materials effectively mitigate systemic chemotherapy side effects. Lastly, while we observed changes in the tumor microenvironment, further investigation is required to elucidate the role of immune cells within this context and explore their interrelationships.

Despite the aforementioned limitations, we have preliminarily demonstrated the anti-tumor efficacy of the GEM and CDDP-loaded mats, thereby establishing a robust foundation for sustained drug delivery to address positive surgical margins and mitigate systemic chemotherapy-induced side effects. Furthermore, exploring the impact of localized chemotherapy delivery using sustained-release materials on the tumor microenvironment will provide novel insights into anti-tumor strategies.

## Conclusions

In summary, we successfully fabricated gemcitabine-cisplatin-loaded nanocomposites from PLA using classical electrospinning technology. By UPLC-MS/MS, we confirmed that the electrospun nanocomposites could significantly improve the drug delivery efficiency and reduce the accumulation of chemotherapeutic drugs in the liver, kidney, and other organs. A mouse bladder cancer model was used to verify the significant anti-tumor effect in vivo and in vitro. Local delivery of chemotherapeutic drugs via slow-release materials can significantly inhibit bone marrow-derived cells and tumor microvascular formation while refinancing CD8^+^ T cells, and NKp46^+^ NK cells into the tumor tissue.

## Data Availability

All data generated or analyzed during this study are included in this article and its Supplementary Material files. Further inquiries can be directed to the corresponding author (YPZ, zhuyunpeng2016@163.com).
